# Functional outcomes and associated factors of cerebral infarction and intracerebral hemorrhage in an area with aging populations in change over time: Evidence from the Akita Stroke Registry

**DOI:** 10.1111/ggi.14598

**Published:** 2023-05-17

**Authors:** Ikue Kudo, Masahiro Sasaki, Akifumi Suzuki, Tatsuya Ishikawa

**Affiliations:** ^1^ Department of Stroke Preventive Medicine Research Institute for Brain and Blood Vessels, Akita Cerebrospinal and Cardiovascular Center Akita Japan; ^2^ Local Incorporated Administrative Agency, Akita Prefectural Hospital Organization Akita Japan

**Keywords:** aging population, cerebral infarction, change over time, functional outcome, intracerebral hemorrhage

## Abstract

**Aim:**

To examine secular change in functional outcomes and associated factors of stroke in a rapidly aging region.

**Methods:**

We retrospectively analyzed cerebral infarction and intracerebral hemorrhage incidence registered cases in the Akita Stroke Registry from 1985 to 2014, divided into three of 10 years each. Functional outcome was defined as good with a modified Rankin scale score of 0–1 and poor with a score of 3–6 at discharge. Mixed effects logistic regression analysis with the location of medical facility as a random effects variable by disease type was used to examine the results.

**Results:**

There were 81 254 eligible patients (cerebral infarction: 58 217, intracerebral hemorrhage: 23 037). Age at onset increased over time in both diseases (cerebral infarction: median [interquartile range] age, 70 [63–77] years in 1985–1994 to 77 [69–83] years in 2005–2014; intracerebral hemorrhage: 64 [56–72] years in 1985–1994 to 72 [61–80] years in 2005–2014). Multivariate analysis showed that the odds ratio associated with good outcomes increased over time for cerebral infarction, and cerebral hemorrhage increased in periods 2 and 3 compared with period 1, but decreased from period 2 to period 3. For cerebral infarction, the odds ratios of prior diabetes associated with poor outcomes decreased over time.

**Conclusion:**

The age at onset increased over time. In cerebral infarction, functional outcomes improved over time, and the association between diabetes and poor outcome declined over time. It was speculated that these results were related to advances in the healthcare system and improved management of vascular risk factors during the study period. Intracerebral hemorrhage improved during the first 20 years, with no apparent improvement thereafter. **Geriatr Gerontol Int 2023; 23: 486–492**.

## Graphical abstract

In the past 30 years, for cerebral infarction, functional outcomes improved over time, and the association between diabetes and poor outcome declined over time. Intracerebral hemorrhage improved during the first 20 years, with no apparent improvement thereafter.

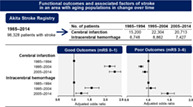

## Introduction

One of the greatest challenges in healthcare today is the global aging population.^1^ Although the incidence of stroke has decreased in developed countries over the past few decades, the absolute number of stroke cases, deaths and lost disability‐adjusted life years has increased, significantly increasing the burden of stroke.^2^ These increases are thought to be due to epidemiological transitions, such as population aging.^3^ Japan has a rapidly aging population, and Akita Prefecture has a particularly high rate of aging. As of 2021, it has the highest aging rate among all prefectures, with 38.5% of its population aged ≥65 years. It has also consistently ranked high in terms of stroke incidence and mortality since the 1950s among all prefectures.

In 1973, Akita Prefecture started a program to register all stroke patients in the prefecture. The project is the largest regional stroke registry in Japan, consisting of all stroke events requiring emergency hospitalization at emergency medical facilities in the prefecture, and all stroke patients and stroke‐related deaths admitted to cooperating medical facilities.^4^, ^5^ To date, >110 000 cases have been registered over >30 years, allowing us to track changes over a long duration. Using long‐term data from the region, analyzing how the aging of the population affects stroke can provide useful insights for other regions with even older populations in the future.

Although population aging is expected to affect functional outcomes of stroke, few studies have examined changes in functional outcomes over time on a regional population basis.^6^, ^7^ In addition, to our knowledge, no studies have examined changes in factors associated with outcomes over time. In the present study, we aimed to examine changes over time in functional outcomes, and factors associated with outcomes over 30 years from 1985 to 2014 using data from the Akita Prefecture Stroke Incidence Registry.

## Methods

### Study population and duration

**Figure 1:**
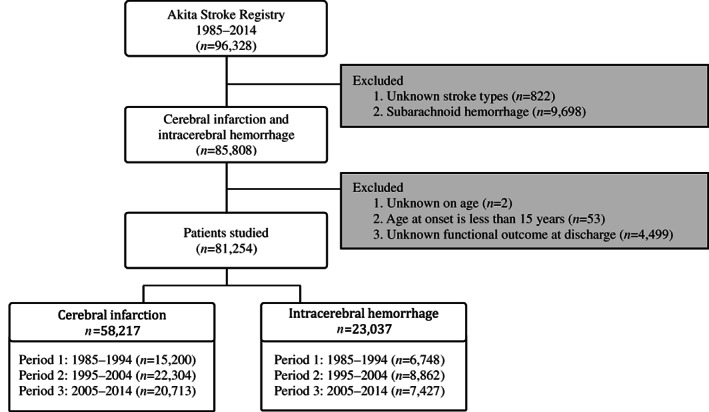
Flow chart of the study design.

The Akita Stroke Registry uses the World Health Organization MONICA stroke diagnostic criteria.^8^ Based on computed tomography or magnetic resonance imaging, patients are classified into three subtypes: intracerebral hemorrhage (ICH), cerebral infarction (CI) and subarachnoid hemorrhage (SAH). In the present study, SAH was excluded from consideration, because its pathophysiology differs from that of CI/ICH. Of 96 328 patients registered during the period covered, 85 808 had a diagnosis of CI or ICH. Of these, we excluded unknown age (*n* = 2) and unknown functional outcome at discharge (*n* = 4 499). Age at onset <15 years was also excluded due to a small number of cases (*n* = 53). Of the remaining 81 254 patients, 58 217 were CI, and 23 037 were ICH (Figure 1). To evaluate secular trends, the period was divided into three 10‐year periods (period 1: 1985–1994, period 2: 1995–2004, period 3: 2005–2014). According to the census, the population of Akita Prefecture was 1 254 032 in 1985 and 1 036 861 in 2014, and the percentage of the population aged ≥65 years was 12.6% in 1985 and 32.6% in 2014.

### Functional outcome measurement

For functional outcomes, we extracted functional outcome assessment data at discharge from the hospital. In this registry, functional outcomes were assessed in the following five categories: “Able to work (ADL 1),” “able to perform all daily activities independently (ADL 2),” “partial assistance needed (ADL 3),” “full assistance needed (ADL 4)” and “death (ADL 5).” These categories were validated by applying them to the modified Rankin scale (mRS),^9^ a measure of post‐stroke life independence widely used in current stroke treatment (ADL 1 was defined as mRS score of 0 to 1, ADL 2 as mRS score of 2, ADL 3 as mRS score of 3 to 4, ADL 4 as mRS score of 5 and ADL 5 as mRS score of 6). As Japan's population ages, the number of people requiring nursing care is a major challenge, and according to a 2019 report by the Ministry of Health, Labor and Welfare, stroke is the second leading cause of requiring nursing care.^10^ The study focused on the need for caregiving and considered an mRS score of 0–1, which corresponds to the state of being able to carry out activities without limitations before the onset of illness, as a good outcome, and an mRS score of 3–5, which indicates the need for some form of assistance, and mRS score of 6, which indicates death, as a poor outcome.

### Clinical parameters

Data on sex, age, clinical characteristics (severity of diseases at onset and history), history of stroke and surgery for ICH were extracted. For severity at onset, data on the level of consciousness at onset were extracted. In the stroke registry, the level of consciousness was classified as “alert,” “somnolent,” “semi‐comatose (not responding to calls but responding to strong pinching)” and “comatose (not responding to pinching or tapping)”. For medical history, data on the presence or absence of a history of hypertension, diabetes, atrial fibrillation and dyslipidemia were extracted. For prior stroke, the percentage of patients with prior stroke (CI, ICH, SAH) was calculated. For surgery, the percentage of patients who had undergone surgery was calculated among the registered cases of ICH.

### Statistical analysis

Baseline characteristics are summarized for each period. Continuous data are reported as the median and interquartile range, whereas categorical data are reported as numbers and percentages. To check for secular trends, we used the Jonckheere–Terpstra trend test for continuous data, and the Cochran–Armitage trend test and χ^2^‐tests for categorical data. Changes in functional outcomes and associated factors over time were analyzed by mixed effects logistic regression analysis, with clustering at the hospital level considered and the hospital as a random effects variable. The hospitals were examined by classifying them into the regional divisions used when examining the medical care system in this prefecture (northern, central, and southern prefectures). The mixed effects model permits improved statistical efficiency given subject‐specific random effects and maximum use of available data even among those with missing data. To assess changes in functional outcomes over time, univariate and multivariate mixed effects logistic regression analyses were carried out, with good and poor outcomes as objective variables. Multivariate models included factors associated with outcomes. Changes over time in the associated factors were analyzed using multivariate analysis, with poor outcome as the objective variable. Significance was defined as a *P*‐value <0.05. Statistical analyses were carried out with Stats version 17 (StataCorp, College Station, TX, USA).

The research protocol conformed to the ethical guidelines of the Declaration of Helsinki, and was approved by the Research Ethics Committee of the Akita Cardiovascular and Cerebrospinal Center, the data management institution (Reception No. 21‐21). The need for informed consent was waived, because this study falls under an exception to the informed consent rule.

## Results

### Baseline of the patients

Baseline data on characteristics, and vascular risk factors and other results for both strokes are presented in Table 1. For both types, there was an increase in the proportion of female patients and an increase in age at onset over time. In addition, the percentage of patients with an “alert” level of consciousness on admission increased over time for both types.

**Table 1:** Baseline characteristics of the patients

Variable	Cerebral infarction				Intracerebral hemorrhage			
	1985–1994	1995–2004	2005–2014	*P*‐value^a^	1985–1994	1995–2004	2005–2014	*P*‐value^a^
No. patients	15 200	22 304	20 713		6748	8862	7427	
Female, *n* (%)	6044 (40)	9799 (44)	9375 (45)	<0.001	2840 (42)	4032 (46)	3443 (46)	<0.001
Median age, years (IQR)	70 (63–77)	74 (67–80)	77 (69–83)	<0.001	64 (56–72)	69 (60–77)	72 (61–80)	<0.001
Age ≥75 years, *n* (%)	5211 (34)	10 339 (46)	12 300 (59)	<0.001	1387 (21)	2786 (31)	3275 (44)	<0.001
Level of consciousness at onset, *n* (%)								
Alert	11 264 (76)	17 249 (79)	16 232 (79)	<0.001	2972 (45)	4241 (49)	3760 (51)	<0.001
Somnolent	2640 (18)	3505 (16)	3417 (17)		2194 (34)	2640 (30)	2244 (31)	
Semi‐comatose	573 (4)	825 (4)	684 (3)		791 (12)	1127 (13)	855 (12)	
Coma	274 (2)	346 (1)	222 (1)		586 (9)	694 (8)	478 (6)	
History at onset, *n* (%)								
Hypertension	7078 (61)	12 007 (55)	11 121 (55)	<0.001	3543 (71)	5526 (65)	4103 (57)	<0.001
Diabetes mellitus	2180 (20)	4719 (22)	4749 (23)	<0.001	588 (12)	1080 (13)	985 (14)	<0.001
Atrial fibrillation	3281 (24)	5519 (26)	5100 (26)	0.005	230 (4)	437 (5)	520 (7)	<0.001
Dyslipidemia	988 (10)	2606 (12)	2500 (12)	<0.001	283 (6)	681 (8)	619 (9)	<0.001
History of stroke, *n* (%)	3459 (23)	5102 (23)	4537 (22)	0.040	1078 (16)	1761 (20)	1562 (21)	<0.001
Surgery, *n* (%)	NA	NA	NA		1385 (21)	1348 (16)	531 (7)	<0.001

^a^ *P*‐values are comparisons between periods.

Abbreviation: IQR, interquartile range; NA, not available.

### Secular changes in functional outcome

**Figure 2:**
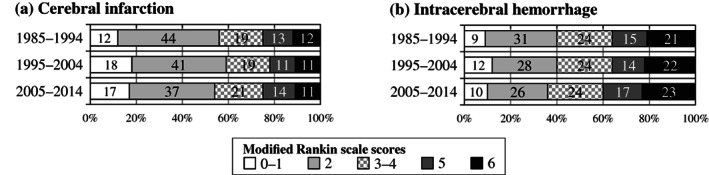
Proportion of functional outcomes at discharge by period.

**Figure 3:**
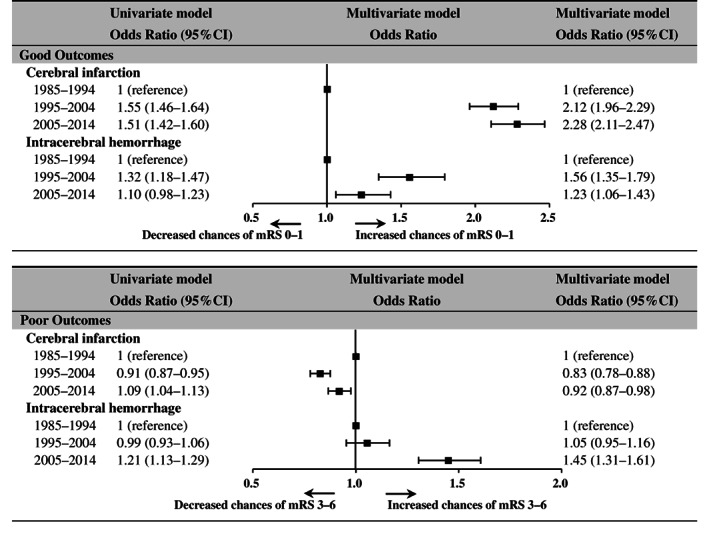
Secular changes in functional outcomes at discharge. Changes over time in good outcomes (modified Rankin Scale [mRS] score of 0–1) and poor outcomes (mRS score of 3–6). Mixed effects logistic regression analysis was used to calculate odds ratios with reference to period 1. Odds ratios for univariate models (left side), odds ratios for multivariate models (right side) and the odds ratios for the multivariate model are shown in a forest plot (center). The multivariate model includes age, female sex, level of consciousness at onset, history at onset (hypertension, diabetes mellitus, atrial fibrillation, dyslipidemia), history of stroke and surgery (intracerebral hemorrhage only).

Figure 2 shows secular changes in the percentage of functional outcomes at hospital discharge. Figure 3 shows the results of a mixed effects logistic regression analysis of the change over time in the proportion of good and poor outcomes. The results of the univariate model and the multivariate model including factors associated with the outcomes are shown, with the first period as the reference period. For good outcomes, CI showed a continuous increase in odds ratios in the multivariate model; ICH increased only in period 2 in the univariate model and both periods in the multivariate model, but more in period 2 than in period 3. For poor outcomes, CI decreased in period 2 and increased in period 3 in the univariate model, whereas the multivariate model showed a similar decrease for both periods; for ICH, both univariate and multivariate models showed no significant change in period 2, and an increase in period 3.

### Secular changes in factors associated with functional outcome

**Figure 4:**
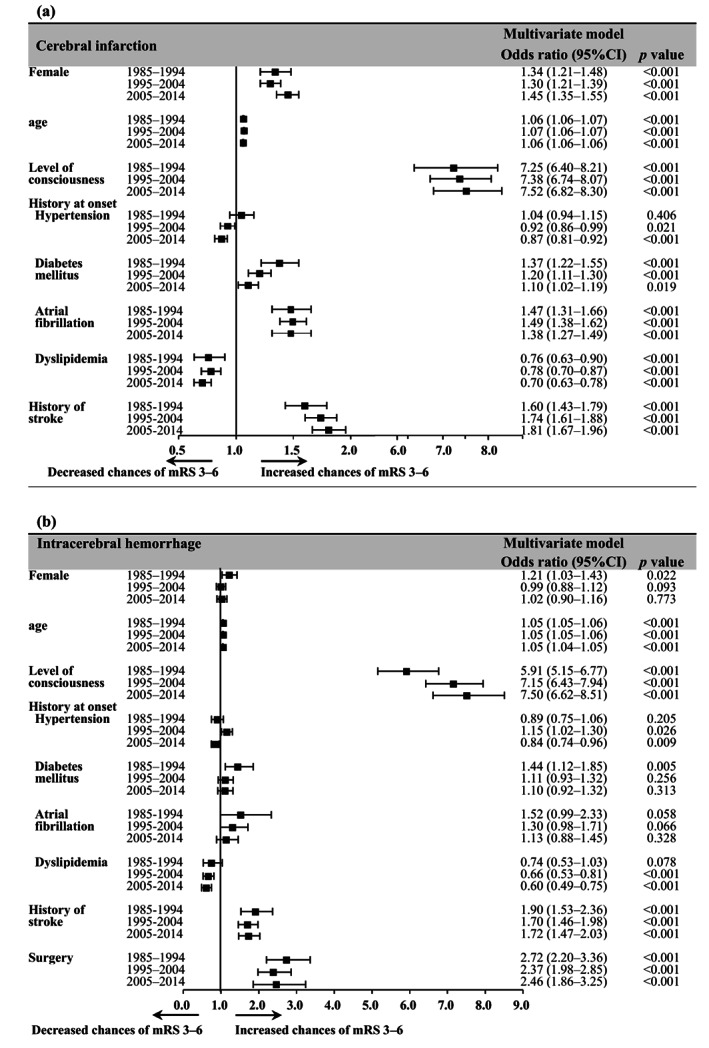
Secular changes in associated factor of functional outcomes at discharge. Changes over time in the association between outcome‐associated factors and poor outcome (modified Rankin Scale [mRS] score of 0–3). Odds ratios from multivariate mixed effects logistic regression analysis. Center is an illustration of odds ratios by forest plot.

The results of the multivariate mixed effects logistic regression analysis of changes over time in factors associated with the poor functional outcome are shown in Figure 4. For both types, age, level of consciousness at onset and history of stroke were associated with poor outcomes, whereas dyslipidemia was associated with reduced poor outcomes. In CI, female sex, diabetes and atrial fibrillation were associated with poor outcomes at all time points, and hypertension was associated with reduced poor outcomes, except for period 1. Surgery in ICH was associated with poor outcomes at all time points. Odds ratios for age did not change over time for both types and were to the same extent at all time points. For CI, odds ratios for diabetes decreased over time.

## Discussion

The present study examined trends in CI and ICH from 1985 to 2014, focusing on changes over time in functional outcomes and factors associated with outcomes. To our knowledge, there have been no studies examining changes over time in factors associated with functional outcomes, and this study is the first to do so.

The age of onset has increased over time in the past 30 years. Other Japanese stroke incidence registry studies^7^, ^11^, ^12^ and French and New Zealand registry studies^13^, ^14^ reported a similar trend. One can speculate that this result is related to demographic changes over the past 30 years. The Hisayama study examined stroke incidence rates by age group and reported that incidence rates increased with age, a trend that remained unchanged from the 1960s to the 1990s.^15^ It is assumed that the population is aging and the absolute number of stroke patients in the older age groups is increasing, and this is likely to be reflected in the present results.

Severity at onset was increasing, with an increasing proportion of mild cases in both types, regardless of adjustment for age and stroke history. This trend has already been noted^7^ and can be assumed to be due in part to the increase in minor strokes since the 1990s, the first period of this study, as imaging devices, such as computed tomography and magnetic resonance imaging, became more widespread and improved diagnostic accuracy. In addition, Akita Prefecture launched the Stroke Prevention Project in 1970, and has been promoting measures against hypertension, salt reduction, health education and public awareness. It can be assumed that these measures have improved awareness of disease and health, led to appropriate management of risk factors for stroke, and that the dissemination of knowledge about stroke symptoms and treatment methods has enabled early consultation, which has led to a decrease in severity at the onset.

For functional outcomes, the multivariate mixed effects logistic regression analysis adjusted by relevant factors showed a continuous increase in the odds ratio for good outcomes in CI, indicating improvement in outcomes over time. In ICH, the odds ratio for good outcomes increased, although not continuously, in periods 2 and 3 compared with period 1, indicating an improvement in outcome. However, for poor outcomes, the odds ratio increased in period 3 compared with the previous two periods, showing a worsening outcome. These results are presented after adjustment for relevant factors, suggesting that there may be factors other than those examined in the present study that influenced the outcome. We believe that the examination of this factor requires consideration of changes in the transition of the medical system surrounding stroke during the period covered by this study. In 2000, period 2 of the present study, rehabilitation during the recovery period became covered by insurance, and the system of intensive rehabilitation took root.

In 2003, the first “Guidelines for Stroke Treatment” were published in Japan, and standard acute phase treatment was implemented. In period 3, thrombolysis with recombinant tissue‐type plasminogen activator was approved for the acute treatment of CI in 2005. These might be reflected in improved outcomes over time in CI and an increased proportion of good outcomes in ICH. In contrast, the increase in poor outcomes in period 3 of ICH can be attributed to several factors. First, there is still no scientifically proven, potent acute therapy comparable with thrombolytic therapy for CI. The second is the impact of antithrombotic. In recent years, there has been an increase in ICH associated with antithrombotic use,^16^ also the association between the use of antithrombotic before the onset of disease and their impact on acute severity and poor outcome.^17^ It is possible that some factors associated with outcomes such as these, which could not be extracted in the present study, might have influenced the results.

Hypertension in periods 2 and 3 in the CI was associated with a reduction in poor outcomes in a multivariate mixed effects logistic regression analysis. Although period 1 did not have a statistically significant association, a comparison of its odds ratios showed a trend toward a continuous decrease during the entire period, suggesting that the association of hypertension with a decrease in poor prognosis might have become stronger over time. Although this result seems counterintuitive, previous studies have reported that taking antihypertensive medications before the onset of illness is associated with good outcomes,^18^ and that poor blood pressure control before the onset of illness is associated with poor outcomes,^19^ which might explain the results of this study. As the dataset in this study only includes the presence or absence of a diagnosis before the onset of the disease, and the details of antihypertensive medication and blood pressure control are not available, this consideration is speculative, it has been reported that both treatments and blood pressure control rates among hypertensive patients in Japan have increased over time during the 30 years from 1980 to 2010. This result coincides with the period covered in the present study, the changes are consistent and the association is predictable.

Diabetes mellitus is associated with poor outcomes in CI and the adjusted odds ratio has decreased continuously, showing that the association with poor outcomes has weakened over time. Numerous studies have shown that diabetes is a poor prognostic factor in CI.^20^ Diabetes, like hypertension, treatment rates have been reported to increase over time.^21^ The results of the present study presumably reflect advances in effective disease management of diabetes.

Dyslipidemia was associated with reduced poor outcomes in both types in the present study. On the association between dyslipidemia and outcome, although various reports have been published to date, a consistent finding is an association between low cholesterol and increased poor outcomes in older adults. For example, a meta‐analysis of observational studies examined the association between total cholesterol and mortality by stroke type and age, and both CI and ICH reported that low total cholesterol was a protective factor in younger patients and a risk factor in older patients.^22^ It has been reported that low cholesterol levels in older adults might be a surrogate for nutritional deficiencies, a precursor to low serum albumin,^23^ and a sign of debilitating disease and, thus, a predisposing factor to increased stroke mortality.^24^ The present study used a population with an increasing number of older adults over time, and it can be assumed that these are the reasons behind the results in which dyslipidemia is associated with a reduction in poor outcomes. Long‐term control of elevated cholesterol levels is associated with a decreased risk of ischemic heart disease and ischemic stroke. The findings of this study should not be interpreted against established statin regimens, but rather as highlighting the risks of undernutrition in older adults.

For both types, age,^25^, ^26^ level of consciousness at onset,^27^ and history of stroke,^25^, ^26^ atrial fibrillation in CI^28^ and surgery in ICH^29^ were associated with poor outcomes, similar to those already reported. The usefulness of surgery for ICH remains a topic of hot debate. It has been noted that the usefulness of surgery depends on the site of bleeding and the amount of bleeding, and that there is no difference in outcome compared with conservative therapy.^30^ Therefore, the results of the present study do not rule out surgery of ICH.

Several limitations of the present study need to be emphasized. First, severity was assessed solely on the level of consciousness. When this project started, the NIHSS, which is now widely used to assess stroke severity, was not yet available, and the level of consciousness criterion commonly used at that time was adopted and has remained unchanged to the present.^31^ In contrast, in reviewing discharge outcomes, it has been reported that there is no difference between the results adjusted for the level of consciousness and those adjusted for mRS score. Therefore, we assessed severity by the level of consciousness, albeit to a limited extent. Second, although the project recorded many variables associated with outcome, it did not measure thrombolytic therapy, antithrombotic drugs before onset, hematoma volume at onset or hematoma increase after onset. Therefore, there was insufficient information to examine in detail changes in functional outcomes over time.

The present study examined changes over time in functional stroke outcomes and related factors of a rapidly aging region. In both disease types, age at onset increased over time. In cerebral infarction, functional outcomes improved over time, and the association between diabetes and poor outcome declined over time. It was speculated that these results were related to advances in the healthcare system and improved management of vascular risk factors during the study period. Intracerebral hemorrhage improved during the first 20 years, with no apparent improvement thereafter.

## Disclosure statement

The authors declare no conflict of interest.

## Data Availability

The data that support the findings of this study are available from the corresponding author upon reasonable request.
